# A financial incentive program to improve appointment attendance at a safety-net hospital-based primary care hepatitis C treatment program

**DOI:** 10.1371/journal.pone.0228767

**Published:** 2020-02-11

**Authors:** Kristen S. Lee, Lisa Quintiliani, Alexandra Heinz, Natrina L. Johnson, Ziming Xuan, Ve Truong, Karen E. Lasser

**Affiliations:** 1 Boston University School of Medicine, Boston, Massachusetts, United States of America; 2 Section of General Internal Medicine, Boston Medical Center, Boston, Massachusetts, United States of America; 3 Boston University School of Public Health, Boston, Massachusetts, United States of America; 4 Clinical Addiction Research and Education Unit, Boston Medical Center, Boston, Massachusetts, United States of America; Middle East Liver Diseases (MELD) Center, ISLAMIC REPUBLIC OF IRAN

## Abstract

**Introduction:**

Hepatitis C (HCV) infection is a significant health threat, with increasing incidence rates in the setting of the opioid crisis. Many patients miss appointments and cannot initiate treatment. We implemented financial incentives to improve appointment attendance in a primary care-based HCV treatment setting.

**Methods:**

We conducted a systems-level financial incentives intervention at the Adult Primary Care HCV Treatment Program at Boston Medical Center which provides care to many patients with substance use disorders. From April 1 to June 30, 2017, we provided a $15 gift card to patients who attended appointments with an HCV treatment provider. We evaluated the effectiveness of the incentives by 1) conducting a monthly interrupted time series analysis to assess trends in attendance January 2016—September 2017; and 2) comparing the proportion of attended appointments during the intervention to a historical comparison group in the previous year, April 1 to June 30, 2016.

**Results:**

327 visits were scheduled over the study period; 198 during the intervention and 129 during the control period. Of patient visits in the intervention group, 72.7% were attended relative to 61.2% of comparison group visits (p = 0.03). Appointments in the intervention group were more likely to be attended (adjusted odds ratio 1.94, 95% confidence interval 1.16–3.24). Interrupted time series analysis showed that the intervention was associated with an average increase of 15.4 attended visits per 100 appointments scheduled, compared to the period prior to the intervention (p = 0.01).

**Conclusions:**

Implementation of a financial incentive program was associated with improved appointment attendance at a safety-net hospital-based primary care HCV treatment program. A randomized trial to establish efficacy and broader implementation potential is warranted.

## Introduction

Approximately 3.5 million persons in the United States are infected with the hepatitis C virus (HCV) [1], with a recent steep increase in new HCV infection in the setting of the ongoing opioid crisis [2]. HCV infection is a frequent cause of end-stage liver disease and mortality in the US, with approximately 18,000 liver deaths in 2016 [3]. With rapid advancements in HCV treatment, achieving sustained virologic response (SVR) is now possible in many patients. However, HCV-infected patients need to follow several steps along a care continuum to achieve optimal health outcomes. These steps include diagnosis of HCV infection, linkage to care with an HCV treatment provider, retention in care for a liver fibrosis staging process, treatment initiation, and monitoring. Several studies have demonstrated that patients are lost to follow-up at several points along the care continuum, and that large gaps exist between current practice and treatment goals for HCV infection [4].

Strategies to address loss to follow-up include utilizing case managers or patient navigators [5, 6] to help guide patients through the treatment process, and co-locating services (e.g. primary care and HCV treatment) [7, 8]. Additional strategies include providing financial incentives for appointment attendance. Financial incentives have been shown to increase attendance rates at counseling sessions among patients with substance use disorders [9]. Among patients with HCV infection and a history of substance use prescribed 12 weeks of a sofosbuvir-containing regimen, a recent pilot study [10] demonstrated successful implementation and acceptance of financial incentives for attendance at clinic appointments, adherence to HCV medication, and achievement of SVR. This study took place at an infectious disease clinic and liver center in an academic medical center. We are unaware of prior studies that have studied the use of financial incentives in primary care-based HCV treatment settings or to incentivize visit attendance prior to the prescription of HCV medication. Therefore, our objective was to evaluate the effectiveness of a financial incentive program to improve appointment attendance at a safety-net hospital-based primary care HCV treatment program.

## Methods

### Design overview

We implemented a systems-level financial incentives intervention in the Adult Primary Care HCV Treatment Program at Boston Medical Center (BMC), the largest safety-net hospital in New England serving many patients with substance use disorder. Program details have been described elsewhere [8]. A multidisciplinary team, including general internists trained to treat HCV (“HCV provider”), a case manager, a pharmacy technician, and a clinical pharmacist staffed the HCV treatment program. Patients attend multiple appointments until treatment completion in the program. These appointments include 1) the initial visit with an HCV provider (MD or NP); 2) liver staging procedures (e.g. transient elastography to estimate level of fibrosis and abdominal ultrasound); 3) the follow-up visit with an HCV provider where a decision to treat is made; 4) visits with a clinical pharmacist for medication teaching, medication adherence counseling and ascertainment of side effects; and 5) a follow-up visit with an HCV provider three months after treatment completion to ascertain SVR, a proxy for cure [11, 12].

All patients referred to the program for HCV treatment who had active infection with a confirmed viral load were eligible to receive financial incentives for attendance at any HCV related appointment during the study time period. We “turned on” the financial incentives intervention from April 1 to June 30, 2017. We chose these months to test the intervention in order to minimize the impact of severe weather (e.g. snow storms) and holidays on appointment attendance. Patients with HIV co-infection and/or decompensated cirrhosis were not eligible to receive incentives as they receive HCV care in specialty settings at BMC. We compared appointment attendance among HCV patients between April 1 and June 30, 2017 (intervention group) and between April 1 and June 30, 2016 (historical comparison group).

### Interventions

We implemented the incentives at three different steps of the HCV care continuum: 1) the initial visit with an HCV provider; 2) the follow-up visit with an HCV provider; and 3) the follow-up visit with an HCV provider 3 months after treatment completion to ascertain SVR (SVR visit). We provided a $15 gift card to Target or CVS for patients who attended any one of these designated appointments with an HCV provider (maximum payout = $45). The HCV program case manager informed patients about the incentive for appointment attendance at the time of scheduling. However, not all patients were aware of the incentives prior to their initial appointment, as some patients did not schedule their appointments through the case manager (they used the call center, front desk, etc.). The case manager ran weekly reports to identify patients scheduled for a qualifying HCV appointment who would be eligible to receive a gift card during the upcoming week. Four HCV providers treated the majority of the patients, and they provided the gift cards directly to their patients after the appointment. For patients seeing other providers, the case manager provided each patient the gift card individually after the visit. Thus, even those who were not notified of the incentives beforehand were still provided a gift card if they attended an eligible appointment during the intervention period. The case manager and HCV providers documented provision of gift cards in the electronic health record (EHR).

We could not reach some of the patients to inform them of the financial incentive prior to their scheduled appointment during the intervention period. However, it is possible that some patients could have heard about the incentive from other patients in our practice. Patients who received the financial incentive for their initial visit would have been aware of the incentive for their follow-up visit, if that visit took place during the intervention period.

### Measures

We extracted EHR data on patients’ age, sex, race, city of residence (Boston vs. other cities), insurance, whether the patient was homeless, whether the patient was treatment naive, had a history of active substance use defined as having used illicit drugs within the 12 months prior to the scheduled appointment, receipt of direct acting antiviral (DAA) used to treat HCV, duration of therapy, whether there was therapy discontinuation, and whether the patient achieved SVR. We also collected data on HCV providers scheduled and whether the HCV provider was the patient’s primary care provider (PCP).

The primary independent variable was whether the visit was scheduled during the time period when patients were eligible to receive financial incentives for appointment attendance. The primary outcome was whether the qualifying HCV appointment was attended. All data were routinely collected during the course of clinical care. A research assistant (VT) performed chart reviews to ascertain appointment attendance and provision of gift cards.

### Analysis

We evaluated the effectiveness of the incentives on promoting appointment attendance by 1) conducting a monthly interrupted time series analysis to account for underlying trends in attendance rates over the entire study period from January 2016 to September 2017 and assess for the impact of the intervention; and 2) comparing the proportion of attended appointments during the intervention period to a historical comparison group of patients scheduled for HCV treatment in the previous year, April 1 to June 30, 2016. We used logistic regression to evaluate missed versus attended appointments and generalized estimating equations to account for clustering of multiple appointments by the same patient. Based on established and hypothesized predictors of appointment attendance in primary care [13, 14], the model included the following potential confounders: patient’s age, sex, race, city of residence (Boston vs. other cities), insurance, whether the patient was homeless and whether the HCV provider was the patient’s PCP. Correlation coefficients for predictors were below 0.60, suggesting absence of collinearity.

### Ethical considerations

An important consideration in designing an incentive program are the legal restrictions on “patient inducement.” Federal law [15] prohibits health care providers from offering items of value to beneficiaries of federal health care programs (e.g. Medicare and Medicaid) if the items are likely to influence the patient’s choice of provider, items or services. Among other safeguards, our study did not offer cash or cash equivalents (e.g. debit card), and we used a nominal incentive amount to minimize its influence on the patient’s choice. The government’s most recent regulatory position is that there is an important difference between *promoting access* to care and *rewarding* patients for accessing care; the latter is disfavored.

The Boston University Medical Campus Institutional Review Board approved the study. We did not obtain consent from patients since this was a system level intervention and the study poses no more than minimal risk to patients. The need for consent was waived by our ethics committee.

## Results

Three hundred twenty-seven patient visits corresponding to 241 unique patients were scheduled over the entire study period; 198 visits during the intervention period (by 149 patients) and 129 visits (by 94 patients) during the control period. About one-third of patients in each group were women (52/149 of intervention patients and 32/94 of control patients). Patients were relatively young, with a mean age of 47 years (standard deviation 13.5). Nearly half (47%) of patients were non-Hispanic whites, and 74% had Medicaid insurance. Thirty-two percent of patients had homelessness documented in the past year. Patients in each group did not differ by age, race, insurance, or housing status. 147/149 (98%) of intervention patients vs. 92/94 (98%) of control patients were treatment naïve, and 43/149 (29%) of intervention patients vs. 24/94 (26%) of control patients had active substance use history defined as having used illicit drugs within the 12 months prior to the scheduled visit. These differences were not statistically significant. More patients in the intervention period (25/87; 29%) received sofosbuvir/velpatasvir than in the control period (3/45; 7%; p = 0.003). Fewer patients in the intervention group were treated with ledipasvir/sofosbuvir (57/87; 66%) vs. control group (37/45; 82%; p = 0.04). Duration of therapy was either 8 or 12 weeks, depending on the clinical scenario. Very few patients in either group received other DAAs (e.g. elbasvir/grazoprevir, sofosbuvir/velpatasvir/voxilaprevir, etc). 82/87 (94%) of intervention patients who received treatment achieved SVR, relative to 45/45 (100%) of control patients. 5/87 (6%) of intervention patients were lost to follow-up relative to none of the control patients. One percent of intervention patients discontinued treatment, vs. 2% of control patients; all of these patients achieved SVR. Between 2016 and 2017, the program expanded and more PCPs were trained to treat HCV. During the control period, appointments were scheduled with 7 HCV providers, and during the intervention period, appointments were scheduled with 12 HCV providers including 5 HCV providers who were scheduled to see the majority of HCV patients (91/94) during the control period.

Table 1 summarizes the demographic characteristics of patient visits according to appointment attendance during the control and intervention periods, respectively.

**Table 1:** Characteristics of patient visits referred to the hepatitis C primary care treatment program and scheduled to see an hepatitis C treatment provider during the comparison period from April 1 to June 30, 2016 and intervention^a^ period from April 1 to June 30, 2017 (n = 327).

Characteristic	n (%)	Attended appointment during comparison period (%)	Attended appointment during intervention period (%)	*P* value
**Total**	327 (100)	79 (61.2)	144 (72.7)	0.03
**Age**				
18–40	124 (37.9)	18 (45.0)	54 (64.3)	0.04
41–64	179 (54.7)	56 (69.1)	77 (78.6)	0.15
65+	24 (7.3)	5 (62.5)	13 (81.2)	0.32
**Sex**				
Female	115 (35.2)	30 (65.2)	54 (78.3)	0.12
Male	212 (64.8)	49 (59.0)	90 (69.8)	0.11
**Race/ethnicity**				
Hispanic (any race)	41 (12.5)	12 (63.2)	18 (81.8)	0.18
Non-Hispanic Black	117 (35.8)	40 (76.9)	50 (76.9)	1.00
Non-Hispanic White	155 (47.4)	23 (43.4)	68 (66.7)	0.005
Other/missing	14 (4.3)	4 (80.0)	8 (88.9)	0.65
**Insurance:**				
Medicaid	241 (73.7)	55 (59.1)	103 (69.6)	0.10
Medicare	58 (17.7)	15 (57.7)	28 (87.5)	0.01
Private	23 (7.0)	8 (88.9)	10 (71.4)	0.32
Other	5 (1.5)	1 (100)	3 (75)	0.58
**Homelessness**^b^ **documented in past year**				
Yes	99 (30.3)	26 (76.5)	43 (66.1)	0.29
No	228 (69.7)	53 (55.8)	101 (75.9)	0.001
**Lives in Boston**				
Yes	200 (61.2)	46 (58.2)	85 (70.2)	0.08
No	127 (38.8)	33 (66.0)	59 (76.6)	0.19
**PCP is HCV provider**				
Yes	73 (22.3)	8 (61.5)	48 (80.0)	0.15
No	254 (77.7)	71 (61.2)	96 (69.6)	0.16

PCP = primary care provider; HCV = hepatitis C virus

^a^Financial incentive program to improve appointment attendance at primary care hepatitis C treatment program

^b^The data taken from the documentation from the patient’s chart in the previous year from the electronic health record

Of all patient visits in the intervention group, 72.7% were attended relative to 61.2% of comparison group visits (p = 0.03). 70.7% of visits during the intervention period were by patients who were informed of the incentive. Among this subgroup, 78% of appointments were attended. Fifty-one percent of patients attended the initial visit in the control period, while 70% of patients attended the initial visit in the intervention period (p = 0.02). Sixty-eight percent of patients attended the follow-up visit in the control period, while 72% of patients attended the follow-up visit in the intervention period (p = 0.72).

In multivariable analyses, patient appointments in the intervention group were more likely to be attended (adjusted odds ratio [aOR] 1.94, 95% confidence interval [CI] 1.16–3.24, p = 0.01). Appointments by patients aged 41 to 64 were more likely to be attended (aOR 2.05, 95% CI 1.09–3.86, p = 0.03) compared to those by patients aged 18 to 40. Appointments by women (aOR 1.84, 95% CI 1.03–3.26, p = 0.04) were also more likely to be attended (Table 2).

**Table 2:** Multivariable analysis^a^ of patient characteristics associated with attendance at a hepatitis C treatment appointment in primary care.

Variable	Odds Ratio (95% CI)	P value
**Age**		
18–40	1.00	
41–64	2.05 (1.09–3.86)	0.03
65+	1.47 (0.41–5.26)	0.55
**Sex**		
Male	1.00	
Female	1.84 (1.03–3.26)	0.04
**Race/ethnicity**		
Black/African-American	1.00	
Hispanic	0.96 (0.40–2.29)	0.93
White	0.49 (0.24–1.02)	0.06
Other	1.97 (0.44–8.85)	0.38
**Insurance**		
Medicaid	1.00	
Medicare	1.29 (0.61–2.73)	0.50
Commercial	1.45 (0.51–4.12)	0.48
Other	1.38 (0.14–13.47)	0.78
**Eligible to receive incentive**^b^	1.94 (1.16–3.24)	0.01
**Homeless**^c^	1.15 (0.66–1.98)	0.63
**Lives in Boston**	0.58 (0.33–1.02)	0.06
**HCV provider is PCP**	1.45 (0.74–2.84)	0.27

CI = confidence interval; HCV = hepatitis C virus; PCP = primary care provider

^**a**^Adjusted for age, sex, race, insurance, eligibility to receive incentive, homelessness, city of residence (Boston vs. other cities), and whether the HCV provider was the patient’s PCP

^b^Fifteen dollar gift card to Target or CVS for patients who attended scheduled appointments with an HCV provider from April 1 to June 30, 2017

^c^The data taken from the documentation from the patient’s chart in the previous year from the electronic health record

**Fig 1:**
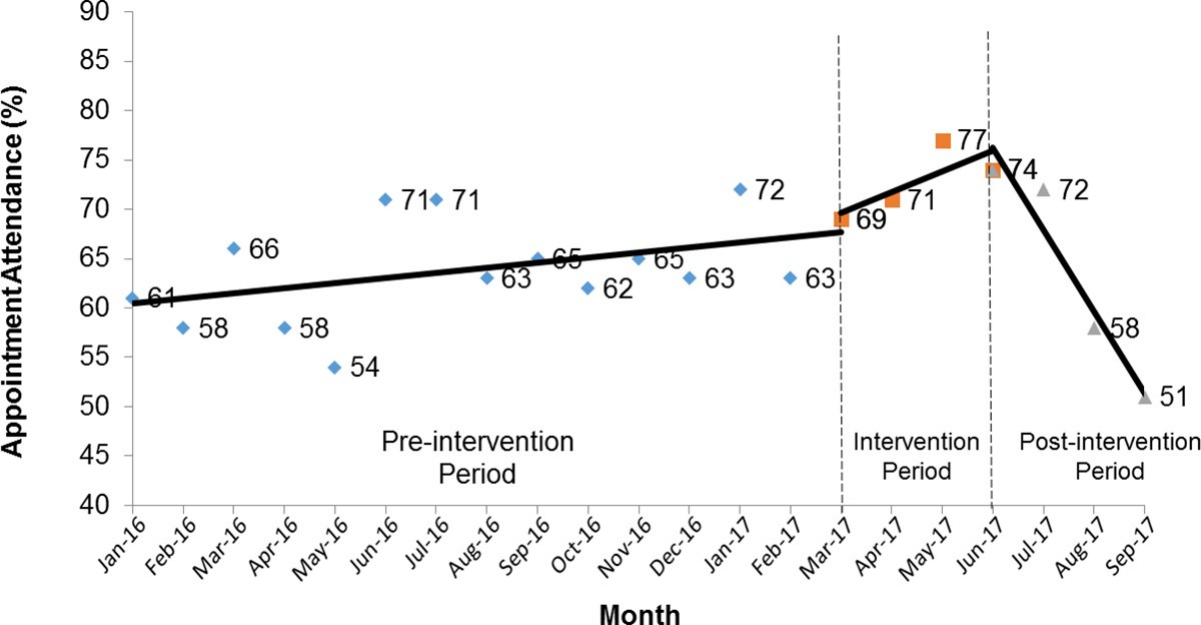
Monthly appointment attendance to HCV provider visits at primary care HCV treatment program from January 2016 to September 2017.

Interrupted time series analysis showed that the intervention was associated with an average increase of 15.4 attended visits per 100 appointments scheduled compared to the period prior to the intervention (p = 0.01). During the three-month post-intervention period, 133 patients had appointments scheduled, and there was a significant downtrend in attendance with a monthly rate of 5.1 additional missed appointments per 100 appointments (p<0.01), even though there was no significant trend over the entire study period (p = 0.11). In the post-intervention period, appointment attendance was lower than that observed in the pre-intervention period, and lower than that observed in the same time period of the previous year (Fig 1).

## Discussion

Our study showed that implementing financial incentives to improve appointment attendance is feasible in a safety-net hospital-based primary care HCV treatment setting. We also showed that financial incentives are associated with improved appointment attendance in this setting, particularly among middle-aged patients and women. Our study extends the work of Wohl et al, who demonstrated that implementation of financial incentives to improve attendance at clinic appointments and to achieve SVR is feasible and acceptable in specialty settings. While Wohl et al focused on patients with HCV infection and a history of substance use who had been prescribed HCV therapy, our study included patients with HCV infection regardless of their substance use history, and intervened on patients prior to receipt of HCV therapy. Many of our HCV treating providers are also waived to prescribe buprenorphine. By increasing appointment attendance, our intervention may also promote patient engagement in medication-assisted therapy for opioid use disorders.

The decrease in appointment attendance after our intervention concluded suggests that the incentives may have been effective. Following the withdrawal of the incentives, patients who were aware of the incentives and had visits scheduled during the post-intervention period may have been less motivated to attend these appointments. Existing evidence from the field of behavioral economics [16] shows a potential for financial incentives to undermine intrinsic motivation in behavior change. This may help to explain why the effect of the intervention was not sustained after the intervention period ended. A future cost-effectiveness analysis is recommended to determine whether it is advantageous to offer a financial incentive program to sustain appointment attendance.

Sustainability of the financial incentive program beyond the funding period has been a challenge. In order to explore this issue in real-world setting, we presented our findings to hospital leadership. We argued that if we can increase appointment attendance and provide treatment to more patients, the hospital will also benefit. Under the US 340b drug pricing program, manufacturers provide medications at significant discounts to 340b covered entities such as safety-net hospitals. Such hospitals are able to dispense these discounted medicines, while Medicaid and private insurers reimburse at full-market price. The 340b program can therefore generate net positive returns, which programs can reinvest in care delivery systems in an effort to improve outcomes [17]. However, providing financial incentives to promote access is a complex and evolving area, and legal or compliance consultation is required before implementing an incentive program, especially outside the clinical trial context.

Our study has several limitations. We did not conduct a randomized controlled trial; we used a historical control group. Therefore it is possible that there were unmeasured differences between the intervention and comparison groups that we did not control for in our analyses. The study was conducted at a single institution, and thus findings may not be generalizable to other settings. In addition, small sample size in some subgroups and relatively short post-intervention period limits our ability to detect an association. In addition, further studies are needed to evaluate if increased attendance rate led to more treatment initiation and SVR.

Implementation of a financial incentive program was associated with improved appointment attendance at a safety-net hospital-based primary care HCV treatment program. A randomized trial of the intervention to establish efficacy and broader implementation potential is warranted.

## References

[1] Edlin BR, Eckhardt BJ, Shu MA, Holmberg SD, Swan T. Toward a more accurate estimate of the prevalence of hepatitis C in the United States. Hepatology. 2015;62(5):1353–63. 10.1002/hep.27978 26171595 PMC4751870

[2] Zibbell JE, Asher AK, Patel RC, Kupronis B, Iqbal K, Ward JW, et al Increases in acute hepatitis C virus infection related to a growing opioid epidemic and associated injection drug use, United States, 2004 to 2014. American journal of public health. 2018;108(2):175–81. 10.2105/AJPH.2017.304132 29267061 PMC5846578

[3] Centers for Disease Control and Prevention. Viral Hepatitis Surveillance—United States, 2016 2016 [Available from: https://www.cdc.gov/hepatitis/statistics/2016surveillance/index.htm.

[4] Yehia BR, Schranz AJ, Umscheid CA, Re VL III. The treatment cascade for chronic hepatitis C virus infection in the United States: a systematic review and meta-analysis. PloS one. 2014;9(7):e101554 10.1371/journal.pone.0101554 24988388 PMC4079454

[5] Trooskin SB, Poceta J, Towey CM, Yolken A, Rose JS, Luqman NL, et al Results from a geographically focused, community-based HCV screening, linkage-to-care and patient navigation program. Journal of general internal medicine. 2015;30(7):950–7. 10.1007/s11606-015-3209-6 25680353 PMC4471023

[6] Coyle C, Viner K, Hughes E, Kwakwa H, Zibbell JE, Vellozzi C, et al Identification and Linkage to Care of HCV-Infected Persons in Five Health Centers-Philadelphia, Pennsylvania, 2012–2014. MMWR Morbidity and mortality weekly report. 2015;64(17):459–63. 25950252 PMC4584550

[7] Ho SB, Bräu N, Cheung R, Liu L, Sanchez C, Sklar M, et al Integrated care increases treatment and improves outcomes of patients with chronic hepatitis C virus infection and psychiatric illness or substance abuse. Clinical Gastroenterology and Hepatology. 2015;13(11):2005–14. e3. 10.1016/j.cgh.2015.02.022 25724704

[8] Lasser KE, Heinz A, Battisti L, Akoumianakis A, Truong V, Tsui J, et al A hepatitis C treatment program based in a safety-net hospital patient-centered medical home. The Annals of Family Medicine. 2017;15(3):258–61. 10.1370/afm.2069 28483892 PMC5422088

[9] Kropp F, Lewis D, Winhusen T. The effectiveness of ultra-low magnitude reinforcers: Findings from a “real-world” application of contingency management. Journal of substance abuse treatment. 2017;72:111–6. 10.1016/j.jsat.2016.06.012 27422452

[10] Wohl DA, Allmon AG, Evon D, Hurt C, Reifeis SA, Thirumurthy H, et al, editors. Financial incentives for adherence to hepatitis c virus clinical care and treatment: a randomized trial of two strategies Open forum infectious diseases; 2017: Oxford University Press. 10.1093/ofid/ofx095 PMC5499638 28695144

[11] Manns M, Pockros P, Norkrans G, Smith C, Morgan T, Häussinger D, et al Long‐term clearance of hepatitis C virus following interferon α‐2b or peginterferon α‐2b, alone or in combination with ribavirin. Journal of viral hepatitis. 2013;20(8):524–9. 10.1111/jvh.12074 23808990

[12] Yoshida EM, Sulkowski MS, Gane EJ, Herring RW, Ratziu V, Ding X, et al Concordance of sustained virological response 4, 12, and 24 weeks post‐treatment with sofosbuvir‐containing regimens for hepatitis C virus. Hepatology. 2015;61(1):41–5. 10.1002/hep.27366 25314116

[13] Lasser KE, Mintzer IL, Lambert A, Cabral H, Bor DH. Missed appointment rates in primary care: the importance of site of care. Journal of Health Care for the Poor and Underserved. 2005;16(3):475–86. 10.1353/hpu.2005.0054 16118837

[14] Pace CA, Gergen-Barnett K, Veidis A, D’Afflitti J, Worcester J, Fernandez P, et al Warm Handoffs and Attendance at Initial Integrated Behavioral Health Appointments. The Annals of Family Medicine. 2018;16(4):346–8. 10.1370/afm.2263 29987084 PMC6037516

[15] Federal Register. Medicare and State Health Care Programs: Fraud and Abuse; Revisions to the Safe Harbors Under the Anti-Kickback Statute and Civil Monetary Penalty Rules Regarding Beneficiary Inducements 2016 [Available from: https://www.federalregister.gov/agencies/health-and-human-services-department. 27992158

[16] Promberger M, Marteau TM. When do financial incentives reduce intrinsic motivation? Comparing behaviors studied in psychological and economic literatures. Health Psychology. 2013;32(9):950 10.1037/a0032727 24001245 PMC3906839

[17] Mitchell GK, Tieman JJ, Shelby-James TM. Multidisciplinary care planning and teamwork in primary care. Medical Journal of Australia. 2008;188(8):S61. 18429739 10.5694/j.1326-5377.2008.tb01747.x

